# Electronic Laboratory Medicine ordering with evidence-based Order sets in primary care (ELMO study): protocol for a cluster randomised trial

**DOI:** 10.1186/s13012-017-0685-6

**Published:** 2017-12-06

**Authors:** Nicolas Delvaux, An De Sutter, Stijn Van de Velde, Dirk Ramaekers, Steffen Fieuws, Bert Aertgeerts

**Affiliations:** 1 Department of Public Health and Primary Care, KU Leuven, Kapucijnenvoer 33, Blok J, PB 7001, B-3000 Leuven, Belgium; 2 Department of Family Medicine and Primary Health Care, Ghent University, Ghent, Belgium; 3 Centre for Informed Health Choices, Norwegian Institute of Public Health, Oslo, Norway

## Abstract

**Background:**

Laboratory testing is an important clinical act with a valuable role in screening, diagnosis, management and monitoring of diseases or therapies. However, inappropriate laboratory test ordering is frequent, burdening health care spending and negatively influencing quality of care. Inappropriate tests may also result in false-positive results and potentially cause excessive downstream activities. Clinical decision support systems (CDSSs) have shown promising results to influence the test-ordering behaviour of physicians and to improve appropriateness. *Order sets*, a form of CDSS where a limited set of evidence-based tests are proposed for a series of indications, integrated in a computerised physician order entry (CPOE) have been shown to be effective in reducing the volume of ordered laboratory tests but convincing evidence that they influence appropriateness is lacking. The aim of this study is to evaluate the effect of order sets on the quality and quantity of laboratory test orders by physicians. We also aim to evaluate the effect of order sets on diagnostic error and explore the effect on downstream or cascade activities.

**Methods:**

We will conduct a cluster randomised controlled trial in Belgian primary care practices. The study is powered to measure two outcomes. We will primarily measure the influence of our CDSS on the appropriateness of laboratory test ordering. Additionally, we will also measure the influence on diagnostic error. We will also explore the effects of our intervention on cascade activities due to altered results of inappropriate tests.

**Discussion:**

We have designed a study that should be able to demonstrate whether the CDSS aimed at diagnostic testing is not only able to influence appropriateness but also safe with respect to diagnostic error. These findings will influence a lager, nationwide implementation of this CDSS.

**Trial registration:**

ClinicalTrials.gov, NCT02950142.

## Background

Laboratory testing is an important clinical act with a valuable role in screening, diagnosis, management and monitoring of diseases or therapies. Thirty percent of patient contacts in primary care result in ordering one or more laboratory tests [1, 2]. With 370 million tests annually, laboratory testing is the most frequent medical activity in Belgium [3]. There is a large variation in the appropriateness of these orders [4–7]. Inappropriate laboratory test ordering has been estimated to be as high as 30% [8, 9]. Besides the burden this poses on health care spending, it also negatively influences quality of care. Inappropriate tests may also result in false-positive results and potentially cause excessive downstream activities. Downstream or cascade activities are those medical acts which result from altered or deviant tests. This phenomenon is often referred to as the Ulysses effect, and it is generally assumed that the effects of inappropriate test ordering are larger on the downstream activities than on the tests themselves [10]. To date, little research has been done on these cascades and the true extent of this Ulysses effect in primary care remains unclear [11].

Education-based interventions, feedback-based interventions and clinical decision support systems (CDSSs) have shown promising results to influence the test-ordering behaviour of physicians and to improve appropriateness [1, 9, 12, 13]. These findings, however, tend not to be generalisable because many studies either focus on very limited indications or measure testing volume rather than appropriateness. *Order sets*, a form of CDSS where a limited set of evidence-based tests are proposed for a series of indications, integrated in a computerised physician order entry (CPOE) have been shown to be effective in reducing the volume of ordered laboratory tests [14, 15]. However, good evidence that the use of order sets aimed at multiple indications improves the appropriateness of laboratory test ordering is still lacking. A barrier to adhering to evidence-based policy is the fear for missing important pathology and the liability this may create [2]. There is currently no evidence that increasing appropriateness of laboratory testing influences morbidity through diagnostic error or delay. The aim of this study is to evaluate the effect of order sets on the quality and quantity of laboratory test orders by physicians. We also aim to evaluate the effect of order sets on diagnostic error and explore the effect on downstream or cascade activities.

## Methods

### Trial design

To evaluate this intervention, we will conduct a cluster randomised controlled trial in Belgian primary care practices. The participants will be general practitioners (GPs) working in primary care practices (PCPs) affiliated to one of three collaborating laboratories in the Leuven, Ghent or Antwerp regions. Currently, these laboratories are starting to implement web-based CPOEs integrated in the electronic health record (EHR) of primary care physicians.

### Participants

PCPs will be considered eligible if all the physicians active in the practice agree to be involved in the study. All physicians will be considered eligible if they:

- Collaborate with either one of three collaborating laboratories: Medisch Centrum Huisartsen (MCH), Anacura or Algemeen Medisch Laboratorium (AML)
- Agree to use the online CPOE for their laboratory test orders
- Use a computerised EHR for patient care
- Have little or no experience in the use of order sets within a CPOE
- Agree to the terms in the clinical study agreement

We will aim our recruitment primarily at GPs with no prior experience in the use of order sets. The rationale for this exclusion criterion is that GPs who already use some form of order sets will not stop doing so if they were to be allocated to the control group. Experience in the use of a CPOE will not be an exclusion criterion as we wish to include GPs with varying experience in the use of IT.

No GPs will be excluded on other grounds than the above. Age, demographics, prior use of a CPOE (without the use of order sets), prior laboratory ordering behaviour, etc. will not be used to exclude eligible GPs. This will provide us with a real-life, representative subset of GPs.

### Interventions

Currently, most laboratory test orders are done through a paper-based system. GPs request or take a blood sample from a patient, order tests manually on a paper form by ticking boxes next to each test, manually add the patient contact detail to the form and send both the form and the test tubes in a plastic bag to the laboratory. Slowly, ambulatory laboratories in primary care have started adopting CPOEs for ordering laboratory tests. CPOEs have several benefits for both laboratories and GPs. They reduce mistakes during the pre-analytical phase, improve timeliness of reporting, reduce mistakes during additional orders on the same sample and reduce the overall turnaround time of samples [16, 17]. The adoption of CPOEs also provides perspectives in providing CDSS through order sets.

We will use two different types of CPOE in our study:

1. Lab Online (Moonchase) implemented at AML and MCH and
2. E-Lab implemented at Anacura

Both systems are online platforms that allow the ordering of laboratory tests and the review of lab results through a web-based interface. They are linked to the EHR and integrate patient contact details through an Extensible Markup Language (XML) message. To date, no patient-specific medical data is shared between the EHR and the CPOE. When a physician initiates a laboratory test order through the EHR, a web browser is opened which allows the physician to order laboratory tests. Currently, users are guided to an overview of commonly used laboratory tests, very much like a paper-based form. In our intervention, physicians will be prompted to enter the indication(s) for ordering laboratory tests through a searchable drop-down menu of common indications or a list of indications which can be selected through tick boxes. Selecting one or more of these indications will prompt a new window which shows the appropriate tests for these indications. In this window, the user will be able to accept the panel without changes, to cancel one or more of the ordered test or to order additional tests. The user will not be restricted in ordering any tests but will be ‘nudged’ in the direction of ordering only the appropriate tests.

We developed a series of order sets based on recommendations available through the EBM*Practice*Net platform [18]. We chose this database because it contains context-specific guidelines on more than 1000 conditions or situations including guidelines from the Flemish College of Family Physicians on the laboratory testing for 20 different indications commonly seen in general practice [19–21]. From these guidelines, we extracted recommendations on 17 common indications for investigation in this trial. These order sets were translated into decision support rules that suggest a panel of recommended tests when the physician records the indications or conditions for testing.

### Implementation

To maximise the effects of our intervention, we planned an implementation strategy using the GUIDES checklist as a reference [22]. We conducted interviews and panel meetings with clinical biologists and information technologists to validate the usability of each order set. We conducted focus group interviews with GPs to identify barriers and facilitators to the use of our intervention in daily practice and used these findings to tailor our intervention where feasible. Currently the intervention is being tested in three GP practices and evaluated for usability and acceptability. At the end of the trial, all participating GPs will be surveyed to identify remaining factors that may have influenced its use.

### Outcomes

#### Primary outcome

The definition of (in)appropriateness is broad and can be interpreted in various ways. In this study, we will use a restrictive definition for appropriateness where a test is considered inappropriate if there is no clear indication for ordering the test [8]. For instance, if, for condition A, five tests are considered appropriate, then all additional tests are considered inappropriate. We will also consider a test inappropriate if it is underutilised or not ordered within a certain time frame for a given condition. For instance, if, for condition A, a test X is considered appropriate if it is ordered once per year, then all missing tests X in that year will be considered inappropriate.

#### Secondary outcomes

Due to the role of the GP as a gateway keeper, caring for a variety of complex patients, he is vulnerable to diagnostic errors [23]. Diagnostic errors are defined as diagnoses that were unintentionally delayed, wrong or missed [24]. In a classification of diagnostic error, amongst the most important reasons for diagnostic error in laboratory testing were the failure or delay in ordering needed tests and ordering of the wrong tests [25]. Despite the vulnerability of primary care for diagnostic error, incidences remain low. Very little reliable figures on diagnostic error due to laboratory testing exist, but several studies estimate it less than 0.1 to 2.5% [26, 27]. An important concern in the interpretation of these results lies in the fact that they are often based on retrospective analyses with hindsight bias [28]. Despite these apparently low figures, fear of diagnostic error (and the related liability) is an important concern for physicians when ordering laboratory tests [2]. Our secondary aim is to demonstrate that improving appropriateness of laboratory testing does not result in more diagnostic errors.

We will define *diagnostic error* as *a diagnosis that was unintentionally delayed (sufficient information was available earlier), wrong (another diagnosis was made before the correct one) or missed (no diagnosis was made)*, in accordance with the definition of diagnostic error by Graber et al. [24]. Our order sets recommend tests for initial testing (when a condition or disease is suspected) or for monitoring (when a condition or disease has been diagnosed, but follow-up for the early detection of potential side-effects of treatment or to monitor the evolution of the condition is warranted). In both situations, the potential for diagnostic error is present. We present some examples of diagnostic error in the Appendix.

Measuring this outcome is a challenge. We will use a stepped approach, combining physicians’ reporting of events, chart review and direct patient interviews of a sample of patients. The rationale for adding patient interviews is that some diagnostic errors do not result in additional visits, further investigations or change in practice and will not be recorded in the EHR. Interviewing the patient is the only way to detect these diagnostic errors. The outcome will be measured as the number of diagnostic errors in each arm.

We will also evaluate the effect of evidence-based order sets on test volume. Inappropriateness is not only a result of overutilisation but also of underutilisation, and improving appropriateness may not necessarily result in reducing test volume [8]. We will measure this outcome to contribute to this discussion. This outcome will be measured as the number of tests ordered in each arm for the 17 indications individually and for all laboratory tests ordered by physicians.

#### Exploratory outcomes

In a subset of 250 laboratory panels, we will review the corresponding patient charts and identify all those activities originating directly from the results of the ordered laboratory tests. We will use the methods by Houben et al. [11] as a guide to identify those laboratory panels that could potentially lead to downstream or cascade activities. We will focus on inappropriate tests in both the control and intervention arm to evaluate the extent of downstream activities and compare the difference in downstream or cascade activities between abnormal and normal results.

### Sample size

Our study involves multiple levels of clustering which are not independent of each other. Lowest on the level of analysis is the individual laboratory test included in our study. We must account for the fact that these tests are not ordered entirely independent of each other in one patient; for instance, a white blood cell count is often ordered together with a white blood cell differentiation. Additionally, there is clustering on the level of the physician. When the same physician orders tests for various patients over time, each of these orders is not independent of the other. For instance, if a physician often requests chloride in patients taking diuretics, then this will probably be so in all patients taking diuretics. Finally, GPs often work in primary care practices. Physicians working in the same practice tend to have similar ordering behaviour implying that the orders made by two physicians in the same practice are not independent of each other. We therefore have a four level clustering comprising test, patient, physician and primary care practice with the following assumptions: multiple study tests per patient, assumed to be 5 per patient; multiple patients per physicians, assumed to be 42 per 3 months if there is 70% use of the online intervention [14]; and multiple physicians per primary care practice (PCP), assumed to be 2.35 [29].

Each of these clustering levels inflates the required sample size [30]. Estimates of intracluster correlation coefficients (ICCs) for process of care measures in primary care are between 0.05 and 0.15 [31, 32]; however, estimates for ICCs regarding the appropriateness of laboratory tests have been shown to vary between 0.04 and 0.288 [33]. To our knowledge, no ICCs have been published for clustering on the various levels observed in our trial; therefore, we have chosen to use a very conservative ICC estimate of 0.2 for each level of clustering in our sample size calculations, probably overestimating the design effect.

A trial with 80% power to detect a 10% difference in appropriateness (in this case, from 70 to 80%) using a significance level of 5% would require 586 tests in both arms. Adjusting for the multi-level clustering inflates this to 7305 tests or 35 physicians in trial lasting 3 months.

We also aim to power our study to its secondary outcome. Assuming 2.5% diagnostic errors in primary care, we calculated that the trial, with an 80% power to detect a non-inferiority of a 1% difference using a significance level of 5%, would need 6032 patients in total. Previous studies have illustrated that in primary care, ICCs for clinical outcomes are lower than those for process outcomes and the ICC for adverse effects to be around 0.025 [34]. Although this may not seem correct, the chances that a physician consistently misses the same diagnosis are less probable than consistently ordering the same test for the same indication. Diagnostic error will more probably lead to a change in practice than over- or underutilisation of a laboratory test. For this outcome, the unit of analysis is the individual patients and not individual tests as for appropriateness, reducing clustering with one level. Assuming a 3-month period in which laboratory tests are ordered for 42 patients, we would need to recruit 290 physicians. Our aim is to recruit 300 physicians, and it is expected that the trial will include around 12,600 patients and a total of 63,000 tests.

### Assignment of interventions

Randomisation of PCPs will be done using an electronic random number generator blinded to the research facility. The research facility will be kept blinded to the allocation, but collaborating laboratories will be able to identify intervention and control PCPs. Allocation will be kept blinded to all PCPs until the start of the study. At the start of the study, GPs will be aware of the intervention and their allocation to either the intervention or control arm; however, patients and research facility will be kept blinded to this allocation during the study and until after the data analysis.

### Data collection, management and analysis

**Fig. 1:**
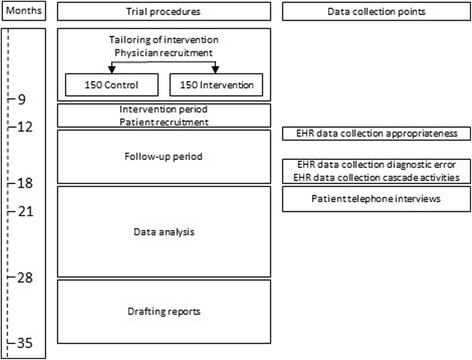
Flow of the study including data collection points, assessments and reports. EHR: electronic health record

The Scientific Institute for Public Health will facilitate data collection and conform to privacy legislation through the Healthdata platform. This platform allows for secure, encrypted and pseudonomysed data transfer from multiple sources into a central data warehouse. Coded data from a single patient can be collected through this system from more than one source. The HD4DP (Healthdata for data providers) tool captures the data from within the EHR or LIS (laboratory information system) and allows the data provider to complement the tool with additional data which is not stored in the EHR in a structured format. The source data remains in the information system of the data provider, and only an excerpt of this source data is transferred to the platform. Data can be collected continuously or in a one-time fashion depending on the resource. All data is transferred through encrypted channels, using highly secured national eHealth encryption algorithms. The research facility can view the coded data using the HD4RES (Healthdata for researchers) tool. The Scientific Institute for Public Health will maintain the data warehouse, including all coded data. The data warehouse is highly secured, and access to the database is only possible through an extranet connection which does not allow full access to the data. Only a small number of members of the Scientific Institute for public Health are authorised to access the full data. Figure 1 illustrates the various phases of the trial including the data collection points.

Baseline data to compare participating physicians will be obtained at the start of the study. This data includes the size of the PCP (how many active physicians), age of the participating physician at the start of the study, sex of the participating physician, number of active years in general practice of the participating physician at the start of the study, average number of tests per laboratory order in the 3 months prior to the study and the level of experience in the use of a CPOE prior to the study.

For comparisons of physician characteristics, comparisons will be made using generalised estimating equations (GEEs), using PCP as the clustering variable. An independent working correlation matrix will be used to account for correlations with the clusters. For continuous variables, an identity link and normal distribution will be used; for binary variables, a logit link and binary distribution; and for categorical variables, a cumulative logit link and multinomial distribution. Means and proportions per group will be estimated from the model. For the comparison of patient characteristics, similar methodology will be used as for physician characteristics but using the physician as the clustering variable.

### Primary outcome analysis

To assess differences between the allocated groups in the proportion appropriate tests, a logistic GEE model will be used: of interest are the marginal proportions, not the individual probabilities of the test to be appropriate.

The logistic GEE model will include the allocated group as a factor and patients as the clustering variable. The effect of the intervention will be expressed as the difference in proportions and will be presented together with its associated 95% confidence interval. The proportion of appropriate tests in the two allocated groups will also be estimated from the GEE model and presented with their 95% confidence intervals.

Appropriateness for the composite of all study tests will be compared between the intervention and control groups. Furthermore, an analysis will be performed that only includes patients who have no indications in addition to the 17 study indications. This additional analysis will correct for an overestimation of inappropriate tests when more than one indication is selected, including indications not under evaluation. These tests would be considered inappropriate even though they could be appropriate according to one of the other indications not being evaluated. The analyses will be performed on all patients from all physicians according to their allocated group.

### Secondary outcome analysis: diagnostic error

The proportion of patients with a missed diagnosis will be analysed by means of a logistic GEE model that includes a factor for allocated group and uses PCP as the clustering variable. An independent working correlation matrix will be used. The proportion of patients with a missed diagnosis and associated 95% confidence intervals will be estimated from the model.

The difference in proportions will be obtained by subtracting the two proportions. The associated standard error will be calculated from the rules for the variance of a difference between two independent estimates. The 95% confidence interval for the difference will be calculated.

The non-inferiority limit for missed diagnoses is 1%, i.e. the intervention will be deemed non-inferior if the difference between the allocated groups (intervention − control) is less than 1%. Therefore, the intervention will be deemed non-inferior if the upper limit of the 95% confidence interval lies below 1.

As for the primary endpoint, the analysis will be performed for all 17 study indications together. An analysis will be performed that only includes patients who have no indications in addition to the 17 study indications.

### Secondary outcome analysis: test volume and cascade activities

The total number of tests and cascade activities will be analysed using a Poisson GEE model that includes the allocated group as the factor in the model and the physician as the clustering variable. No offset will be used. The number of tests per patient for each group will be estimated from the model and presented together with their associated 95% confidence intervals. The effect of the intervention will be presented as the ratio between the two numbers with its 95% confidence interval. Statistical significance will be assessed at a significance level of 5%.

## Discussion

The effects of decision support have shown to be modest and often inconsistent [35, 36]. Some of these CDSSs have been implemented in settings not receptive to these systems, lacked essential technical qualities or standards, suffered from usability issues or were not trustworthy enough [37, 38]. In this study, we used the insights in the mechanisms that influence effectiveness of CDSS to tailor our intervention where feasible. Moreover, the potential for improvement is substantial with high rates of inappropriateness. We developed 41 different order sets (including sets with optional tests according to specific patient characteristics) for a total of 18 different indications.

The results of this study will reflect the true effect of a CDSS in regular practice because we aim to include a large sample of GPs with varying degrees of experience in the use of a CPOE. We realise that an important feature critical to the success of our CDSS is the degree in which physicians use the laboratory CPOE. In the tailoring strategy for our study, we focussed on this issue and aimed to improve the order sets in a fashion that would make the care processes involved in ordering laboratory tests more efficient. We will investigate any shortcomings of our intervention at the end of the trial to further improve its efficiency and effectiveness. Additionally, we have designed a study that should be able to demonstrate whether the CDSS aimed at diagnostic testing is not only able to influence appropriateness but also safe with respect to diagnostic error. More attention is being focussed on patient safety as an important goal for health care, and recommendations have been made on implementing safety systems [39]. Our study will contribute to the discussion on how CDSS systems can assist not only in process of care but also in safe care. These findings will influence a lager, nationwide implementation of this CDSS. These plans are facilitated by the fact that most laboratories in Belgium use the same software for their CPOE.

## Appendix: examples of diagnostic error

### Example of diagnostic error in initial testing (wrong test)

A physician suspects that his patient, who presented with a swollen knee, suffers from an acute attack of gout. He decides to test his patient for plasma uric acid to confirm his suspicion. Plasma uric acid levels are elevated, apparently confirming his suspicion, and he prescribes the patient a non-steroidal anti-inflammatory drug. Several days later, the patient returns with fever, increased swelling of the knee and intensified pain. The physician aspirates some synovial fluid and notices pus in the sample. The patient did not suffer from a gout attack but from a septic arthritis. A diagnostic aspiration of synovial fluid would have diagnosed the condition earlier.

### Example of diagnostic error due to not testing

A female patient consults with general fatigue. The GP, knowing that the patient has a stressful life, explains the fatigue as a reaction to this stress. He considers laboratory testing not relevant in this case. A few months later the patient is diagnosed with hypothyroidism.

### Example of diagnostic error due to superfluous testing

A young adult patient consults because of decreased power during his weekly football training. The GP performs a laboratory test which shows increased Epstein-Barr antibodies (EBV-IgG). The GP ascribes the complaints to a recovering mononucleosis infection and misses the real reason for the complaints, which is overtraining. EBV-IgG indicates an old infection and has no diagnostic value in this case but led the GP to a wrong conclusion.

### Example of diagnostic error in follow-up testing

A patient consults his physician for a yearly check-up of hypertension for which he has been prescribed a thiazide diuretic. The physician checks his blood pressure and finds it well controlled. He confirms the treatment and prescribes refills without performing any laboratory tests. Several weeks later, the patient consults the emergency room because of a sudden syncope. In the hospital, the physicians note that he suffers from a hypokalaemia due to the thiazide treatment. This is an example of a missed diagnosis that should have been made during the yearly check-up of the hypertension treatment.

## Abbreviations

AML: Algemeen Medisch Laboratorium
CDSSs: Clinical decision support systems
CPOE: Computerised physician order entry
EHR: Electronic health record
GP: General practitioner
HD4DP: Healthdata for data providers
HD4RES: Healthdata for researchers
MCH: Medisch Centrum Huisartsen
PCP: Primary care practice
XML: Extensible Markup Language
